# MicroRNA let-7b inhibits cell proliferation via upregulation of p21 in hepatocellular carcinoma

**DOI:** 10.1186/s13578-020-00443-x

**Published:** 2020-07-01

**Authors:** Li Hui, Fang Zheng, Yuan Bo, Ma Sen-lin, Li Ai-jun, Zhou Wei-ping, Zhang Yong-jie, Yin Lei

**Affiliations:** 1 The Department of Dermatology, Changhai Hospital, Second Military Medical University, Shanghai, 200438 China; 2 The Second Department of Biliary Surgery, Eastern Hepatobiliary Surgery Hospital, Second Military Medical University, Shanghai, 200438 China; 3 The Third Department of Hepatic Surgery, Eastern Hepatobiliary Surgery Hospital, Second Military Medical University, Shanghai, 200438 China

**Keywords:** Hepatocellular carcinoma, MicroRNA, Let-7b, Cell proliferation, p21

## Abstract

**Background:**

Hepatocellular carcinoma (HCC) is one of the most malignant tumor types and has a high incidence and mortality. Many miRNAs play important roles in the development of HCC. Identification of these miRNAs and their targets is increasingly urgent for a better understandingof miRNA function in both physiological and pathological contexts. Many studies have shown that the expression of let-7 is often downregulated in the process of tumorigenesis, suggesting that let-7 may participate in this process as an oncogene.

**Methods:**

Immunochemistry staining was used to observe the expression of let-7b in HCC tissues. A CCK-8 assay was employed to detect the role of let-7b in the proliferation of HCC cells. The cell cycle of HCC cells was examined by flow cytometry. BALB/c nu/nu mice were used to detect the tumorigenesis potential of HCC cells; western blot and real-time PCR were employed to observe the expression of p21 in HCC cells.

**Results:**

In our previous studies investigating HCC tissue samples obtained from the national tissue samples bank of liver cancer in Eastern Hepatobiliary Surgery Hospital, we found one abnormal expression of miRNA (let-7b), which was significantly downregulated in HCC tissue. In the current work, we studied the relationship between let-7b and HCC to potentially provide invaluable information for developing novel therapeutic strategies for treating HCC. Based on our findings, let-7b expression was absent in HCC tumors, and its lower expression was associated with poor prognosis of HCC. In further experiments, we found that let-7b inhibited HCC cell proliferation through upregulation of p21.

**Conclusion:**

The results of our study suggested that let-7b might inhibit the proliferation of HCC cells by upregulating p21.

## Background

Hepatocellular carcinoma (HCC) is the sixth most common malignancy worldwide and has persistently increasing rates of both incidence and mortality [1]. Tumor resection and liver transplantation are the major therapeutic strategies for HCC patients. Despite recent advances in surgical techniques and perioperative management, the prognosis of HCC remains poor [2]. Therefore, it is important to recognize the potential mechanism of HCC occurrence and development, which can contribute to developing effective approaches for the early diagnosis and therapy of HCC.

MicroRNAs (miRNAs) are noncoding RNAs of approximately 18–25 nucleotides that act as negative regulators of numerous target mRNAs. Over the past few years, miRNA profiling studies have indicated that many miRNAs are abnormally expressed in HCC tissue and affect the initia-tion and progression of HCC [3, 4]. The miRNA let-7 plays a vital role in tumor suppression in many cancers, including esophageal squamous cell carcinoma, lung cancer and prostate cancer [5–7]. In addition, let-7 is also associated with poor prognosis in HCC patients [8]. Let-7 has multiple subtypes (a, b, c, d, e, f, and g). Extensive evidence suggests that let-7 functions as a tumor suppressor by targeting multiple oncogenes and that a reduction in let-7 is strongly associated with increased tumorigenicity and poor prognosis in patients [7, 9]. The aberrant expression of let-7 is also known to contribute to the development and progression of HCC.

In our previous studies investigating HCC tissue samples from the national tissue samples bank of liver cancer in Eastern Hepatobiliary Surgery Hospital, we found one abnormal expression of miRNA (let-7b), which was significantly downregulated in HCC tissue. However, the role of this abnormal expression of miRNA in the HCC development process is not very clear. In the current work, the function and molecular mechanisms of let-7b in HCC were studied to potentially help better elucidate the molecular mechanism of the development of liver cancer and explore potential therapeutic targets to provide experimental evidence.

## Materials and methods

### Patients and specimens

In this study, HCC tissues and adjacent nontumor tissues were obtained from 94 patients with HCC who received a hepatectomy from March 2010 to October 2010 in Shanghai Eastern Hepatobiliary Surgery Hospital. All patients provided informed consent before enrollment in the study, which was approved by the Ethics Committee of Shanghai Eastern Hepatobiliary Surgery Hospital. Tissue specimens were collected and stored at − 80 °C until required for analysis. Follow-up was completed on July 1, 2014. The follow-up period was defined from the date of surgery to the date of patient death or the last follow-up point.

### Cell culture

The human L02 liver cell line and hepatoma cell lines SMMC-7721, BEL-7402, HepG2, QGY-7703, and Hep3B were used in the current study. The cells were cultured in endotoxin-free Dulbecco’s modified Eagle’s medium with 10% fetal bovine serum (Gibco Life Technologies, Carlsbad, CA, USA). The primers and siRNA sequences involved in this study are shown in Table 1.

**Table 1:** Primers and siRNA sequences

Name	Primer sequence
β-actin F	5′-CAGCAAGCAGGAGTATGACG-3′
β-actin R	5′-GAAAGGGTGTAACGCAACTAA-3′
U6 F	5′-GTGCTCGCTTCGGCAGCACATATAC-3′
U6 R	5′-AAAAATATGGAACGCTCACGAATTTG-3′
p21 F	5′-AAACTAGGCGGTTGAATGAG-3′
p21 R	5′-AAAGGAGAACACGGGATGAG-3′
P21 siRNA (sense)	5′-GACCAUGUGGACCUGUCACdTdT-3′
P21 siRNA (antisense)	5′-GUGACAGGUCCACAUGGUCdTdT-3′
NC siRNA (sense)	5′-AAUUCUCCGAACGUGUCACdTdT-3′
NC siRNA (antisense)	5′-GUGACACGUUCGGAGAAUUdTdT-3′
Let-7b mimics	5′-UGAGGUAGUAGGUUGUGUGGUU-3′
NC mimics	5′-UUGUACUACACAAAAGUACUG-3′
Let-7b F	5′-GTACTGAGGTAGTAGGTTGT-3′
Let-7b R	5′-GTGCAGGGTCCGAGGT-3′
Anti-Let-7b	5′-ACACGAATTCAACCACACAACCTACTACCTCATATACAACCACACAACCTACTACCTCAACATCAACCACACAACCTACTACCTCATCTTCAAACCACACAACCTACTACCTCAGGATCCACAC-3′

### Real-time PCR

Total RNA was isolated from tissues using TRIzol (Invitrogen, Carlsbad, CA) following the manufacturer’s instructions. Single-stranded cDNA was synthesized using a Prime Script RT reagent Kit (Takara, Kyoto, Japan). Standard RT-PCR was conducted using a SYBR Green PCR Kit (Applied BI) according to the manufacturer’s instructions. The reactions were incubated in a 96-well plate at 95 °C for 10 min, followed by 40 cycles of 95 °C for 15 s and 60 °C for 1 min. U6 was used as an endogenous control.

### Cell transfection

A let-7b mimic and a negative control were obtained from Guangzhou RiboBio Co., Ltd., (Guangzhou, China). Cells were plated at 45–50% confluence. The let-7b mimic and negative control were transfected into the human hepatoma HepG2 and QGY-7703 cells using transfection reagent INTERFER in (Polyplus) according to the manufacturer’s instructions. The expression level of let-7b was quantified using RT-PCR 24 h after transfection.

### Cell counting kit-8 (CCK-8) assay

ACCK-8 assay was performed to determine cell proliferation. A total of 5 × 10^3^ cells in 96-well microplates were cultured at 37 °C for 24 h, 48 h and 72 h. The number of viable cells was measured by adding 10% cell counting kit-8 (CCK-8) assay according to the manufacturer’s instructions.

### Cell cycle analysis

For cell cycle analysis, cells were washed with cold PBS three times and digested to obtain a monoplast suspension. Cells were fixed with 70% ethanol overnight at 4 °C, washed with PBS and incubated with propidium iodide staining buffer (BD Biosciences) for 30 min at 37 °C. Flow cytometric analysis was carried out using a FACS Calibur flow cytometer (BD Biosciences) and CellQuest software. Cell cycle distribution was analyzed using FlowJo software.

### In vivo tumorigenicity experiments

Six-week-old male athymic BALB/c nu/nu mice were obtained from Shanghai Experimental Animal Center, Chinese Academy of Science. Mice were maintained under pathogen-free conditions. A total of 5 × 10^6^ cells transfected with the let-7b mimic or negative control were injected subcutaneously into the left back of the mice. At the end of 4 weeks, the mice were sacrificed. The experiments were performed in accordance with the institutional animal welfare guidelines of the Shanghai Eastern Hepatobiliary Surgery Hospital.

### miRNA target predictions

RNA22 prediction software (http://starbase.sysu.edu.cn/index.php) was used to predict let-7 family members that could potentially bind to the mRNA of p21.

### Western blot analysis

Western blot was performed according to a previous study [10]. Mouse monoclonal anti-p21 antibody (1:1000; Cell Signaling Technology, Danvers, MA, USA) and a mouse monoclonal anti-β-actin antibody (1:1000; Sigma-Aldrich, St. Louis, MO, USA) were used.

### Statistical analysis

All of the experiments were repeated at least three times. Analysis of variance was performed using GraphPad Prism 5.0 (GraphPad Software). Quantitative data were expressed as the mean ± SD for each experiment. Significance between groups was determined by Student’s t test. Statistical analysis was performed using SPSS 20.0 for Windows (SPSS Inc., Chicago, IL); differences between categorical variables were assessed by the Chi square test or Fisher’s exact test. A log-rank test was used to compare patient survival between subgroups. P < 0.05 was considered statistically significant.

## Results

### Let-7b expression in HCC tissues and the relationship with the prognosis of HCC patients

**Fig. 1:**
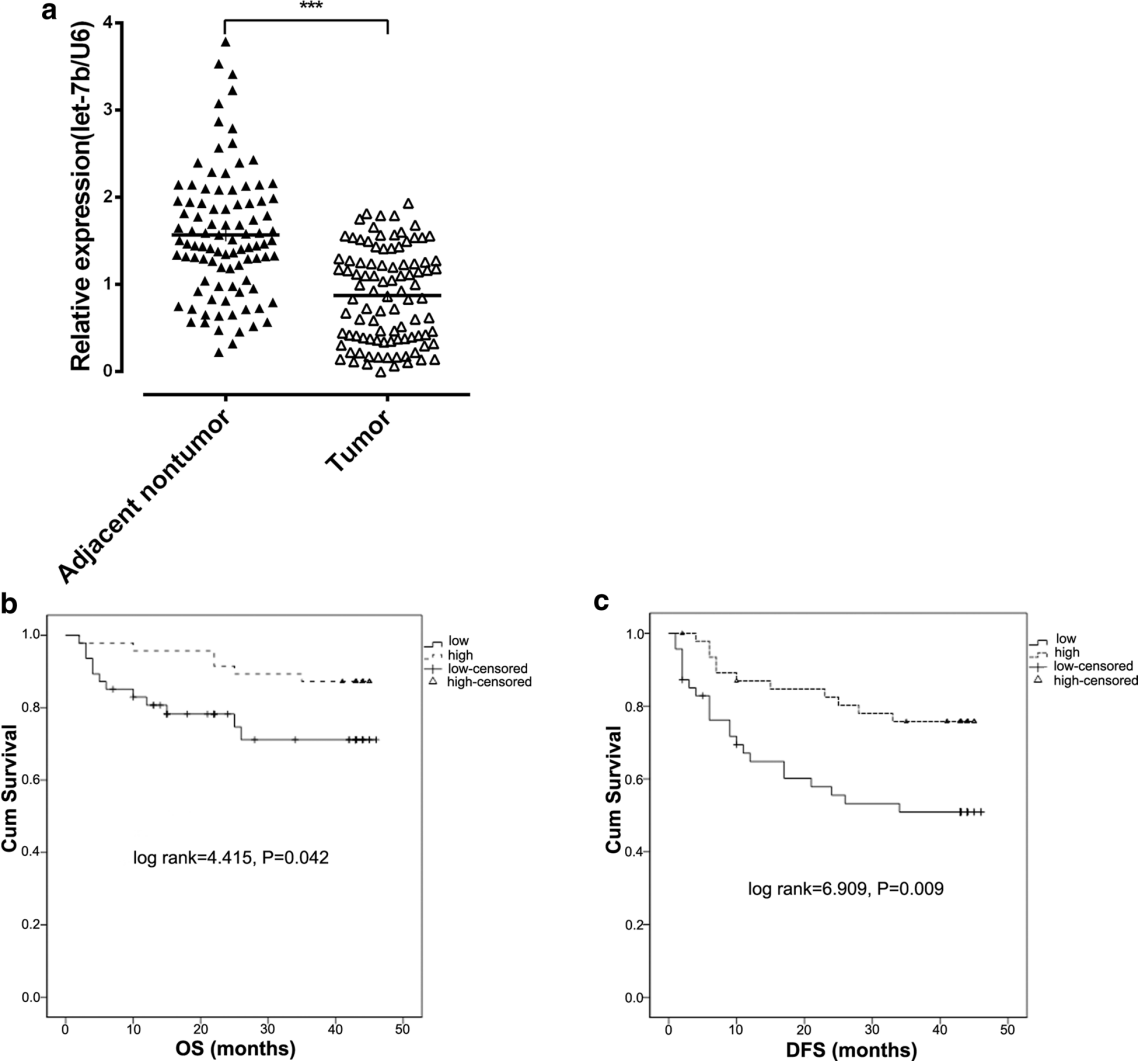
Let-7b expression in HCC tissues. **a** RT-PCR was performed to detect let-7b expression in adjacent nontumor and tumor tissue of HCC patients. Data from three replicates are shown as the means (± SD). ***P < 0.001. **b**, **c**. Overall survival and estimated cancer-free survival according to the expression of let-7b in 94 cases of HCC (Kaplan–Meier method)

To determine the expression of let-7 in the early stage of HCC, RT-PCR analysis was employed to assess the expression of let-7b in HCC and corresponding nontumor (NT) tissues. As shown in Fig. 1a, let-7b expression was significantly downregulated in HCC tissues compared with adjacent control tissues (P < 0.001). We next analyzed the prognosis of HCC patients with low and high let-7b levels. According to the results, 94 HCC cases were divided into two groups: the high expression group (n = 47) and the low expression group (n = 47). The clinical features were analyzed between the two groups (Table 2). HCC patients in the low expression group had either worse overall survival (P = 0.009; Fig. 1b) or worse cancer-free survival (P = 0.042; Fig. 1c) than those in the high expression group. The results indicated that let-7b might be a cancer suppressor gene in HCC.

**Table 2:** Clinical baseline of HCC patients

Characteristics	Let-7b low (n = 47)	Let-7b high (n = 47)	*P* value
Age (years) (mean ± SD)	49.8 ± 9.3	51.8 ± 11.6	0.354
Gender (Male/Female)	40/7	39/8	0.778
HBsAg (positive/negative)	40/7	39/8	0.778
Cirrhosis (positive/negative)	26/21	19/28	0.148
Child–Pugh classification(A/B)	46/1	45/2	0.554
AFP (ng/mL)			0.626
≥ 400	35	37	
< 400	12	10	
Biggest tumor diameter (cm) (mean ± SD)	6.8 ± 4.6	6.0 ± 4.8	0.363
Blood transfusion (positive/negative)	8/39	6/41	0.562
Microvascular invasion (positive/negative)	18/29	17/30	0.831
Portal vein invasion (positive/negative)	4/43	2/45	0.399
Tumor capsule (positive/negative)	9/38	10/37	0.797
BCLC stage			0.779
A	14	17	
B	29	27	
C	4	3	

### Let-7b decreased the proliferation of HCC cells

**Fig. 2:**
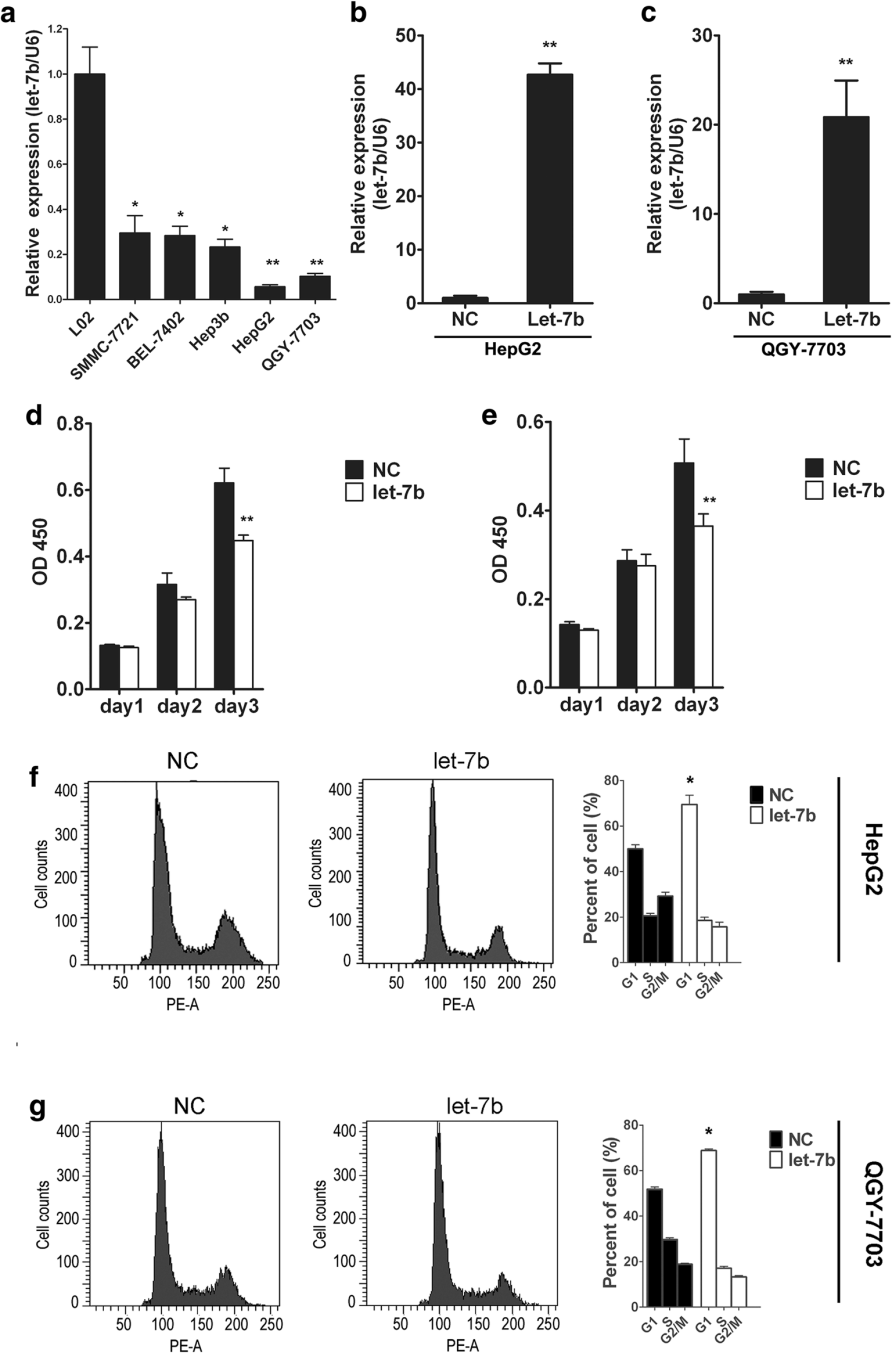
Let-7b overexpression decreased the proliferation of HCC cells. **a** RT-PCR was performed to detect let-7b expression in normal liver cells and HCC cell lines. Data from three replicates are shown as the means (± SD). *P < 0.05, **P < 0.01. **b, c** Let-7b expression was tested by RT-PCR when HepG2 and QGY-7703 cell lines were transfected with negative control (NC) and let-7b mimics (Let-7b). Data from three replicates are shown as the means (± SD). **P < 0.01. **d, e** CCK-8 assay was used to detect cell proliferation of HepG2 and QGY-7703 cell lines treated with negative control (NC) and let-7b mimics (Let-7b). Data from three replicates are shown as the means (± SD). **P < 0.01. **f, g** Flow cytometric analysis was performed to test the cell cycles of HepG2 and QGY-7703 cell lines treated with negative control (NC) and let-7b mimics (Let-7b). Data from three replicates are shown as the means (± SD). *P < 0.05

We then detected let-7b expression in hepatocytes (L02) and HCC cell lines (SMMC-7721, BEL-7402, HepG2, QGY-7703, Hep3B). The results showed that compared with normal liver cells, let-7b expression was significantly downregulated in HCC cells, especially in QGY-7703 and HepG2cells (Fig. 2a). To investigate the role of let-7b in the proliferation of HCC cells, the HepG2 and QGY-7703 cell lines were used for transfection with let-7b mimics. Then, RT-PCR was employed to examine let-7b expression, and the data showed that let-7b was significantly upregulated in the HepG2 and QGY-7703 cell lines after transfection (Fig. 2b, c). Furthermore, let-7b overexpression inhibited the proliferation of HCC cells (Fig. 2d, e). In further experiments, we found that let-7b inhibited cell proliferation and blocked cells through G1/S detection (Fig. 2f, g).

### Let-7b inhibited the tumorigenesis potential of HCC cells

**Fig. 3:**
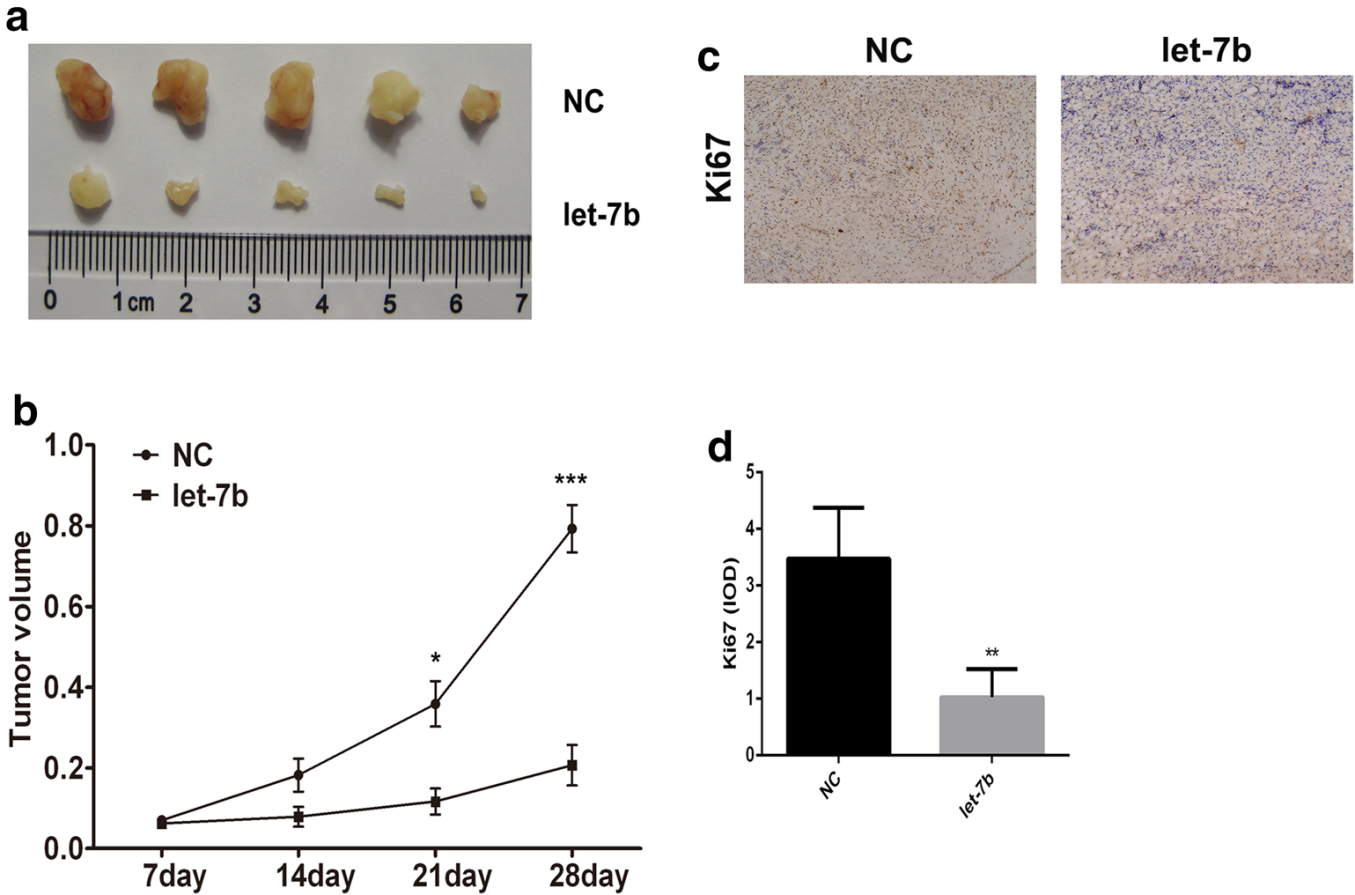
Let-7b overexpression inhibited the tumorigenesis potential of HCC cells. **a**The tumorigenicity of HepG2 cells transfected with let-7b mimics was detected. Pictures were taken at different times after subcutaneous injection. **b** Quantitative analysis of tumor volume in each group. Data from three replicates are shown as the means (± SD). **P < 0.01, ***P < 0.001. **c** Immunohistochemical staining was employed to detect the expression of Ki67 in HCC tissues. **d** The expression of Ki67 in HCC tissues was evaluated and the data was measured by IOD

Next, the effect of let-7b on tumorigenic characteristics was determined by in vivo tumor development experiments. HepG2 cells with let-7b upregulation displayed a significant decrease in activity of tumorigenicity in comparison to the control (Fig. 3a, b), which suggested the inhibitory effect of let-7b on tumorigenesis of HCC cells. We also detected the proliferation status of HCC cells by observing the expression of Ki67. The result showed that compared with control group, let-7b could effectively inhibit the expression of Ki67 in HCC cells (Fig. 3c). In addition, we also inhibited the expression of let-7b in L02 cells and detected the proliferation of L02 cells. As shown in Additional file 1: Figure S1, inhibition of let-7b could lead to slightly higher proliferation in L02 cells Table 1.

### Let-7b suppressed HCC cell proliferation by upregulating p21

**Fig. 4:**
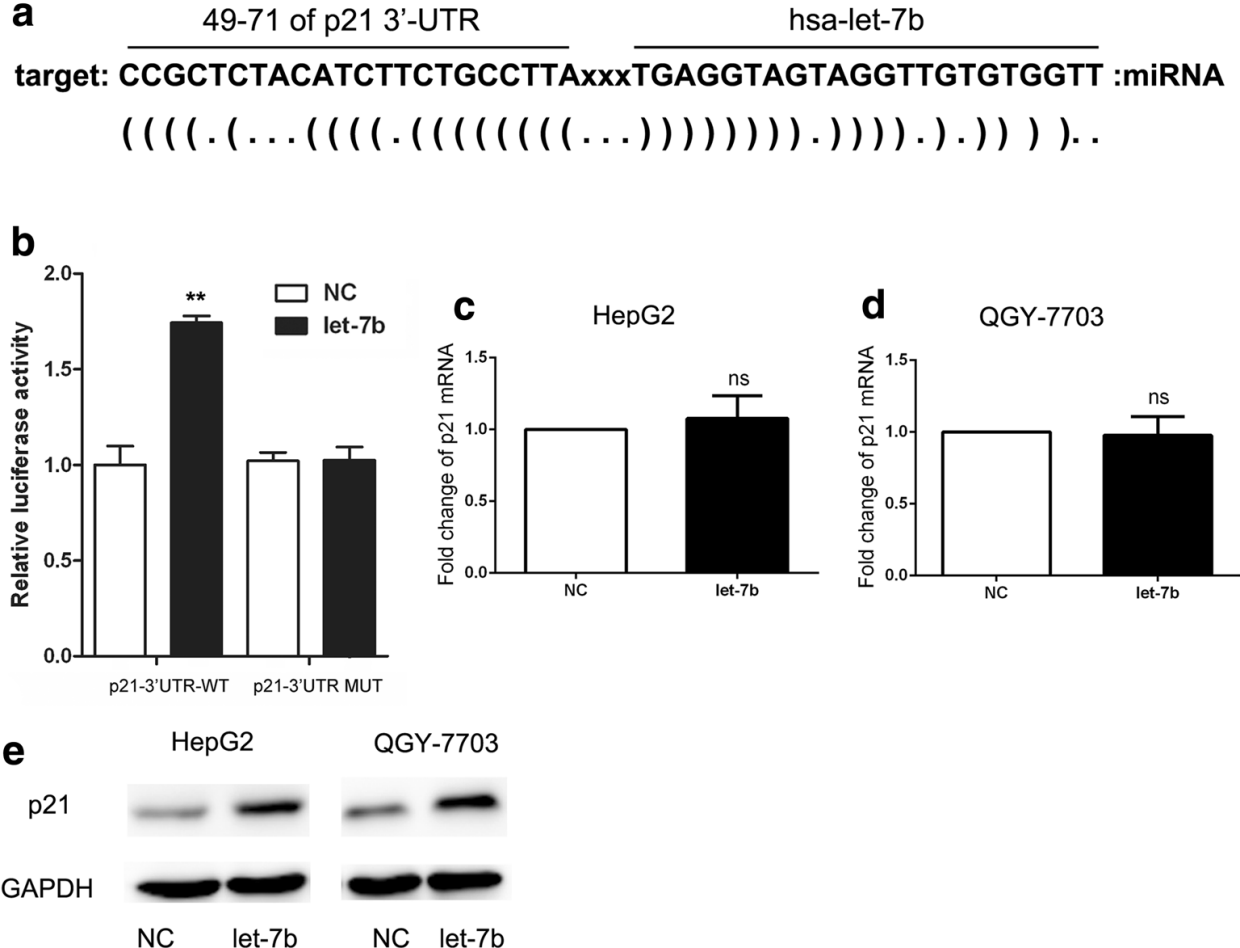
P21 was predicted to be a targeted gene of let-7b. **a** Targeted gene analysis was performed by RNA22. The results showed that the 3′-UTR of the p21 gene contained binding sites for let-7b. **b** p21 expression was determined by luciferase reporter gene assay. **c, d** p21 expression was determined by RT-PCR when HepG2 and QGY-7703 cell lines were transfected with negative control (NC) and let-7b mimics (Let-7b). Data from three replicates are shown as the means (± SD). **P < 0.01, ***P < 0.001. E. p21 expression was determined by western blot when HepG2 and QGY-7703 cell lines were transfected with negative control (NC) and let-7b mimics (Let-7b)

To determine the mechanism of let-7b underlying the inhibition of cell proliferation, we performed an in silico screening using RNA22 [11]. We examined the expression of p21 in hepatocytes (L02) and HCC cell lines (SMMC-7721, BEL-7402, HepG2, QGY-7703, Hep3B). The results showed that there were no significant differences in the mRNA levels in the cell lines, and at the protein level, the expression of p21 in L02 cells was slightly higher than in other HCC cell lines (Additional file 2: Figure S2).We found that the 3′-UTR of the P21 gene contained binding sites for let-7b (Fig. 4a). Therefore, p21 was predicted to be one of the target genes of let-7b. Subsequently, plasmids pMIR-p21 3′UTR-WT and pMIR-p21 3′UTR-MUT were constructed. Let-7b can target 3′-UTR of the p21 gene, and let-7b mimics could upregulate the expression of the fluorescent reporter gene (Fig. 4b). The results showed that the let-7b mimics upregulated the p21 protein levels but not the associated mRNA level (Fig. 4c–e), which means that let-7b targeted the p21 gene and upregulated the expression of the p21 gene at the posttranscriptional level. Furthermore, to detect whether p21 was involved in the inhibition of let-7b-induced cell proliferation, siRNA was used to knockdown p21 expression.

**Fig. 5:**
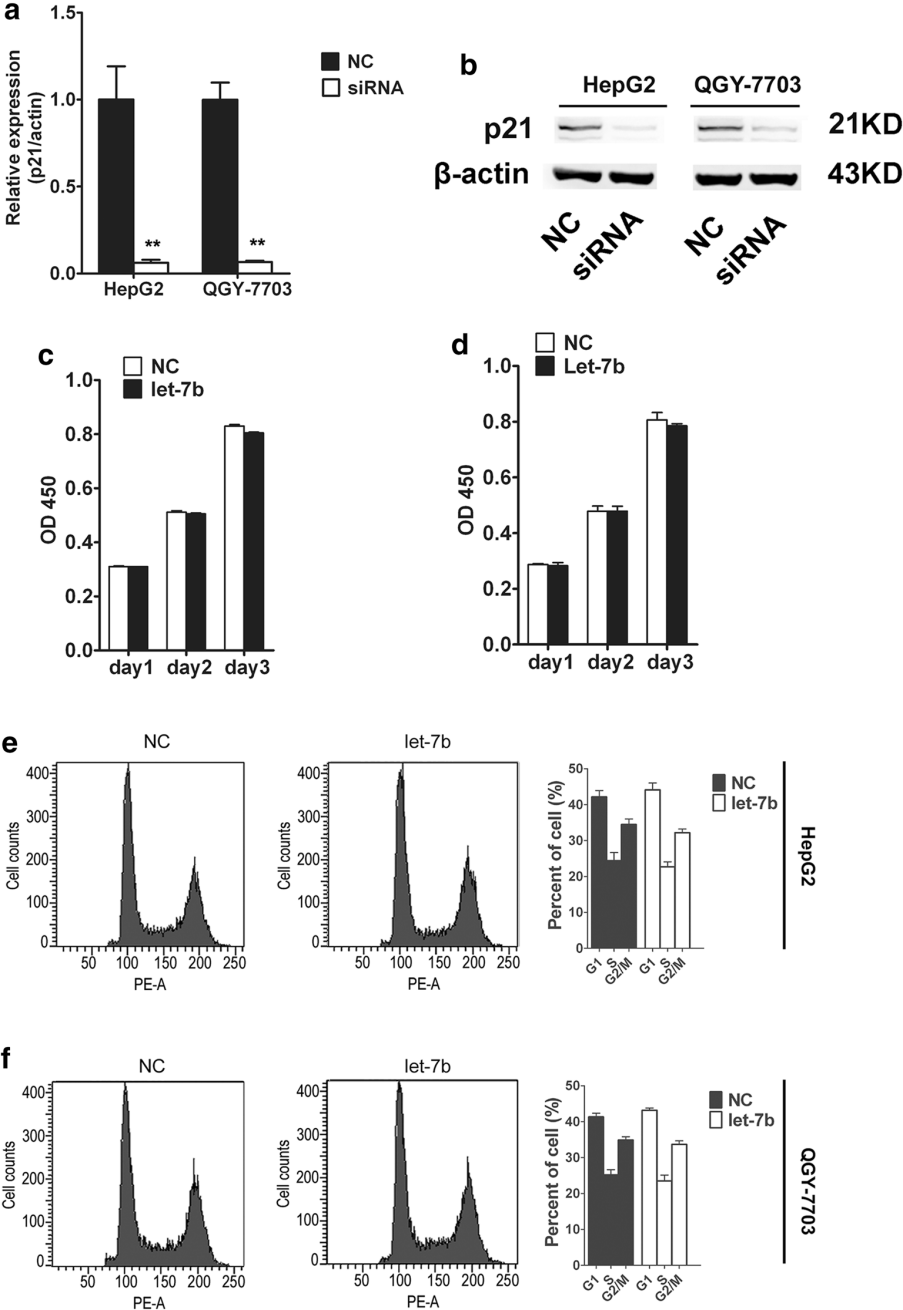
Let-7b suppress HCC cell proliferation by upregulating P21. **a** HepG2 and QGY-7703 cell lines were transfected with p21 siRNA, and p21 mRNA levels were examined by RT-PCR. Data from three replicates are shown as the means (± SD). **P < 0.01. **b** HepG2 and QGY-7703 cell lines were transfected with p21 siRNA, and p21 protein levels were examined by western blot. **c, d** CCK-8 assay was used to detect cell proliferation of HepG2 and QGY-7703 cell lines treated with p21 siRNA. Data from three replicates are shown as the means (± SD). **P < 0.01. **e, f** Flow cytometric analysis was performed to test the cell cyclesof HepG2 and QGY-7703 cell lines treated with p21 siRNA. Data from three replicates are shown as the means (± SD). *P < 0.05

Next, we investigated the role of p21 in the inhibitory effect of let-7b on HCC cell proliferation. The RT-PCP and western blot data confirmed the inhibitory effect of the siRNA on p21 expression (Fig. 5a, b). Then, let-7b-mediated cell proliferation was examined when p21 was silenced. After p21 siRNA treatment, let-7b overexpression could not inhibit cell proliferation or block cells through G1/S detection (Fig. 5c–f). The above results indicated that let-7b suppressed the proliferation of HCC cells by promoting p21 expression.

### Correlation of let-7b and p21 expression in HCC patients

**Fig. 6:**
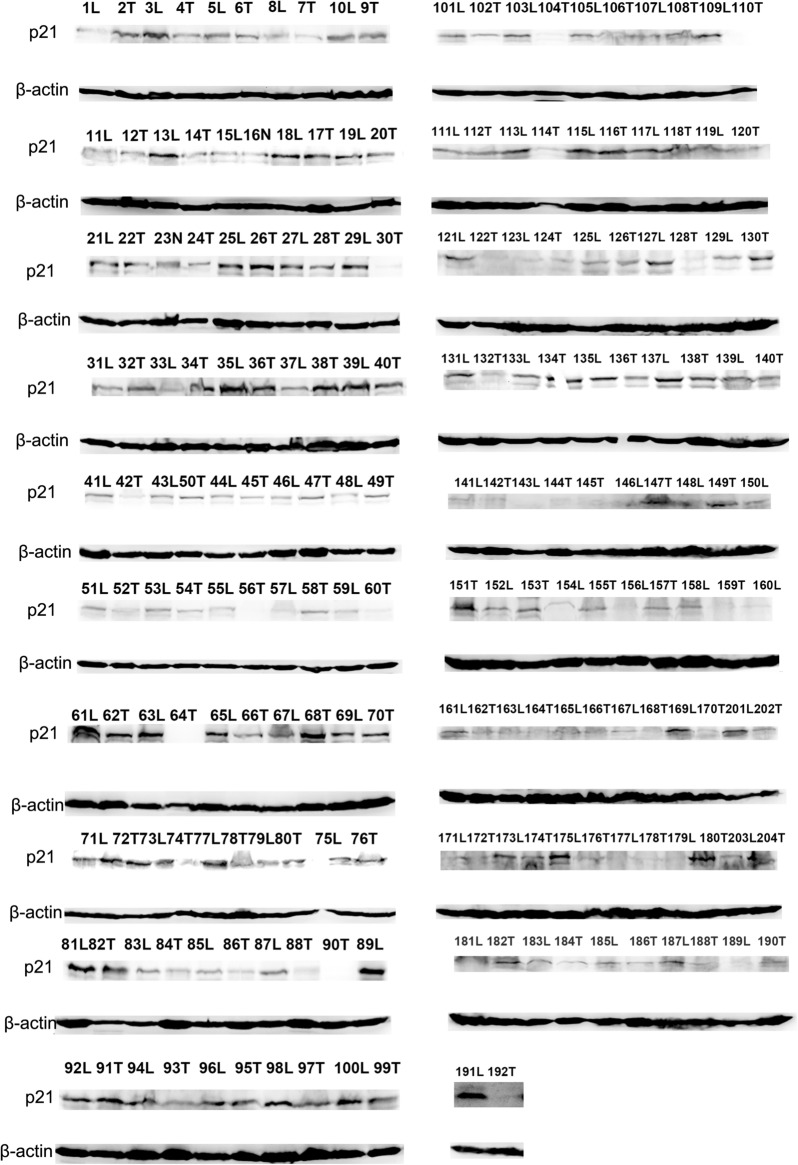
P21 expression in HCC patients. Western blot was performed to detect p21 expression in adjacent nontumor and tumor tissue of HCC patients

**Fig. 7:**
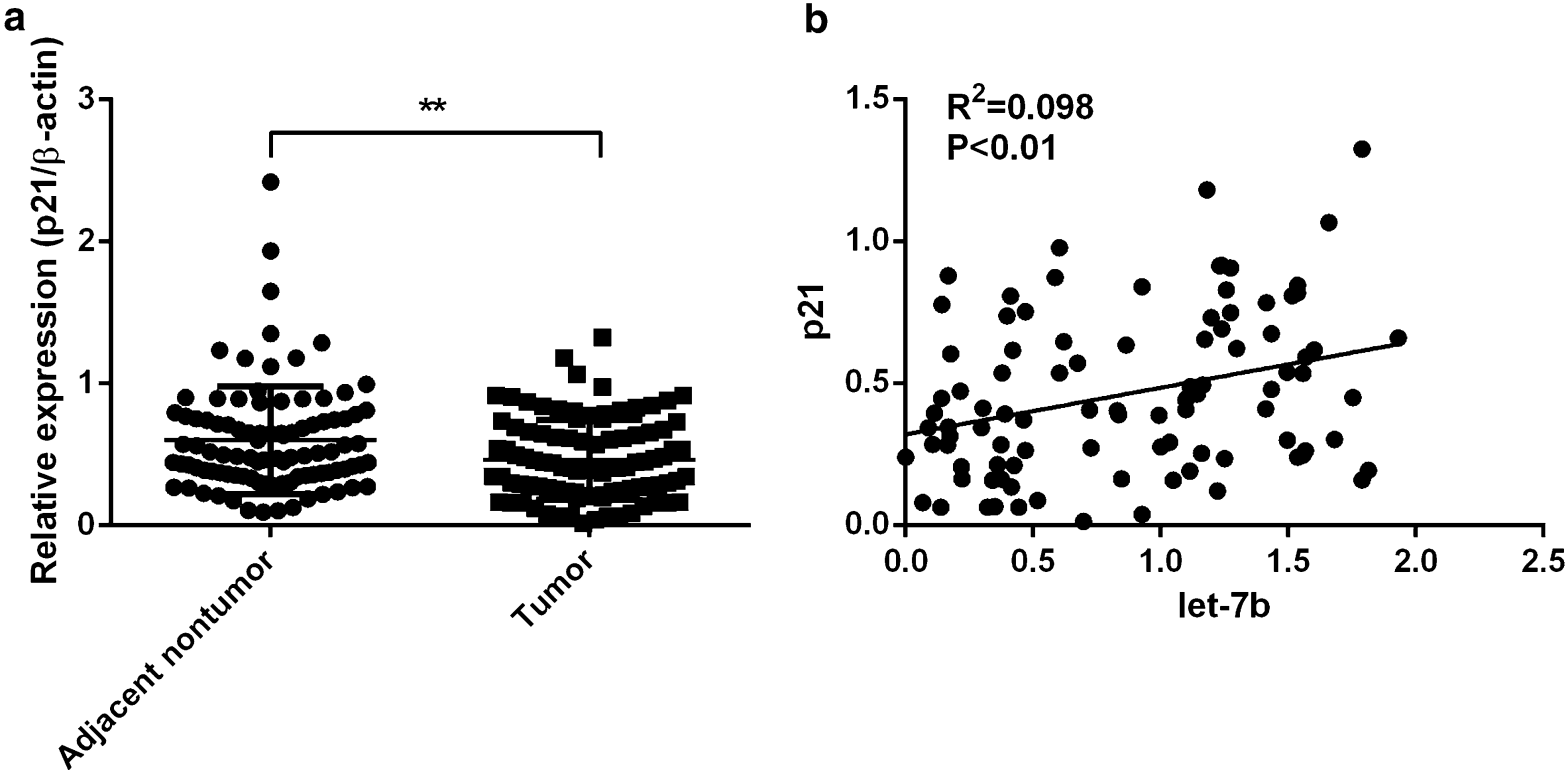
Correlation of let-7b and p21 expression in HCC patients. **a** Quantitative analysis of p21 expression in adjacent nontumor and tumor tissue of HCC patients. **P < 0.01. **b** Expression of p21 in tumor tissues showed a positive correlation withlet-7b

We next investigated the correlation of let-7b and p21 expression in HCC tissues. Western blot was performed to detect p21 expression in HCC tissues. P21 levels were clearly downregulated in HCC tissues and adjacent control tissues. We found that p21 levels were obviously downregulated in HCC tissues (Figs. 6, 7a). Furthermore, the correlation of let-7b and p21 expression was analyzed, and p21 downregulation was correlated with the downregulation of mature let-7b (Fig. 7b). These results suggest that p21 is potentially involved in let-7b-regulated tumorigenesis [12, 13].

## Discussion

Although numerous studies on HCC have been carried out for years, the molecular mechanisms of its genesis, development and metastasis have yet to be fully elucidated. It has been reported that microRNAs played an important role in suppressing the development of hepatocellular carcinoma [14]. Growing evidence has demonstrated that let-7 acts as a tumor suppressor in various cancers. In the present work, we studied the relationship between let-7b and HCC, which may provide invaluable information for developing novel therapeutic strategies for HCC. Based on our findings, let-7b expression was lost in HCC tumors, and its lower expression was correlated with poor prognosis of HCC. In further experiments, we found that let-7b inhibited HCC cell proliferation through upregulation of p21.

The expression of the let-7 series of miRNAs is particularly abundant in normal cells. However, this expression is often significantly downregulated during the process of tumorigenesis, which indicates that let-7 may act as a tumor suppressor gene in the development of tumors [15–17]. Let-7 is a family consisting of 13 members located on nine different chromosomes. Previous studies on the miRNA profile of HCC clinical samples showed that the expression of let-7 was widely downregulated in HCC patients. For example, the expression of let-7 g was downregulated and closely associated with HCC metastasis and prognosis [18, 19]. The expression of let-7b, -7g,-7i, -7d, -7a, -7c and -7e has also been reported to be downregulated in Huh7 cells when compared with normal hepatocytes [20]. We examined the expression of let-7b in 94 cases of HCC and paracancerous tissues. The results demonstrated that the expression of let-7b was downregulated in 83.0% of HCC tissue. In addition, we found that compared with normal hepatocytes, let-7b expression was significantly downregulated in HCC cells, especially in QGY-7703 and Hep3B. These results indicate that the physiological function of let-7b may inhibit the growth of liver cancer cells.

A gain-of-function experiment was carried out in the QGY-7703 and Hep3b cell lines with let-7b mimics. Our results showed that the proliferation of QGY-7703 and Hep3b cells was significantly inhibited when cells were transfected with let-7b mimics. Cell cycle analysis showed that let-7b overexpression blocked G1/S phases. It has been reported that let-7 g is a tumor suppressor gene that acts as a cell proliferation inhibitor in HCC cells [19]. Overexpression of let-7a was shown to inhibit lung cancer cell growth and pancreatic cancer cell proliferation [21, 22]. Therefore, it is not surprising to find another let-7 family member, let-7b, which also possesses tumor suppression characteristics.

It has been reported that let-7b is associated with a variety of diseases and tumors, including acute leukemia, cardiac hypertrophy, cervical cancer, colon cancer, lung cancer, melanoma, myeloma, neuroblastoma, thyroid cancer, prostate cancer and ovarian cancer [22–27]. However, these results only indicated the abnormal expression of let-7b in these diseases; the potential mechanism is still unknown. The development of tumors is closely associated with the proliferation of tumor cells, and the cell cycle plays an important role in controlling cell proliferation.

miRNAs regulate gene expression at the posttranscriptional level by binding to target mRNAs, leading to mRNA degradation or translation inhibition. We ran software to predict the target gene of let-7b. The results suggested that p21, which has been reported to be a key factor in regulating cell proliferation and the cell cycle, might be the target gene of let-7b. We found that the 3′-UTR of the *p21* gene contained binding sites for let-7b. p21 plays an important role in cellcycle control and cell proliferation. The expression of p21 is regulated by gene transcription, mRNA stability, translation, protein stability and posttranslational modifications [28]. Our study also demonstrated a strong correlation between the inhibitory effect of let-7b on the proliferation of QGY-7703 and Hep3b cells and p21.

P21 is an important member of the cyclin-dependent kinase inhibitor family and cyclin, CDK and CDKI constitute a regulatory network that regulates the cell cycle [29]. Cyclin positively controls cell proliferation, and CDKI plays a negative regulatory role in cell proliferation. The p21 protein binds to and inhibits the cyclin-CDK2 or -CDK4 complex to block cells from advancing from G1 phase to S phase. At the same time, P21 can inhibit the proliferation of proliferating cell nuclear antigen (PCNA), thereby inhibiting the synthesis of DNA, detaching the nucleus from the cell cycle, stopping the differentiation of cells, and participating in DNA damage repair. In addition, p21 also plays an important role in various physiological processes, such as apoptosis, cell growth and cell senescence. Several studies have indicated low expression or deletion of p21 in liver cancer, while high expression of p21 leads to cell cycle arrest in hepatocarcinoma and ultimately induces apoptosis of hepatoma cells [30–32].

We investigated whether let-7b can target the p21 gene. The results showed that let-7b could target the 3′UTR region of the p21 gene and upregulate the expression of p21. To further validate the results we obtained, we also observed the coexpression of let-7b and p21 in liver cancer tissue samples. We found that the expression of let-7b and p21 in liver cancer tissues was positively correlated. We also interfered with the expression of p21 in hepatocarcinoma cells. The results showed that the inhibitory effect of let-7b on the proliferation of HCC cells was attenuated by interference with the expression of p21. Cell cycle detection also found that the effect of let-7b on G1/S blocking also disappeared in HCC cells when the expression of p21 was inhibited by siRNA. These results once again confirmed that let-7b can target the p21 gene in hepatoma cells and exert a tumor suppressor effect by regulating the expression of the p21 gene.

## Conclusions

Taken together, the present study suggested that let-7b plays an important role in inhibiting HCC tumorigenesis and progression. This inhibitory ability may be carried out via the upregulation of p21. Further investigations will be necessary to fully reveal the underlying molecular mechanism, yet let-7b is undoubtedly a novel potential target worth further investigation in treating HCC.

## Supplementary information

**Additional file 1: Figure S1.** Inhibition of let-7b on the proliferation of L02 cells. A sponge assay was used to downregulate the expression of let-7b in L02 cells. A CCK-8 assay was employed to detect the proliferation of L02 cells. *P < 0.05.

**Additional file 2: Figure S2.** The expression of let-7b in hepatocyte and HCC cell lines. RT-PCR and western blot were performed to detect let-7b expression in normal liver cells and HCC cell lines. Data from three replicates are shown as the means (± SD).

## Data Availability

Not applicable.

## Abbreviations

HCC: Hepatocellular carcinoma
miRNAs: MicroRNAs
