# Supplementary figures and images for: MicroRNA let-7b inhibits cell proliferation via upregulation of p21 in hepatocellular carcinoma
Source: Cell Biosci. 2020 Jul 1;10:83. doi: 10.1186/s13578-020-00443-x (PMC7329548; doi:10.1186/s13578-020-00443-x)

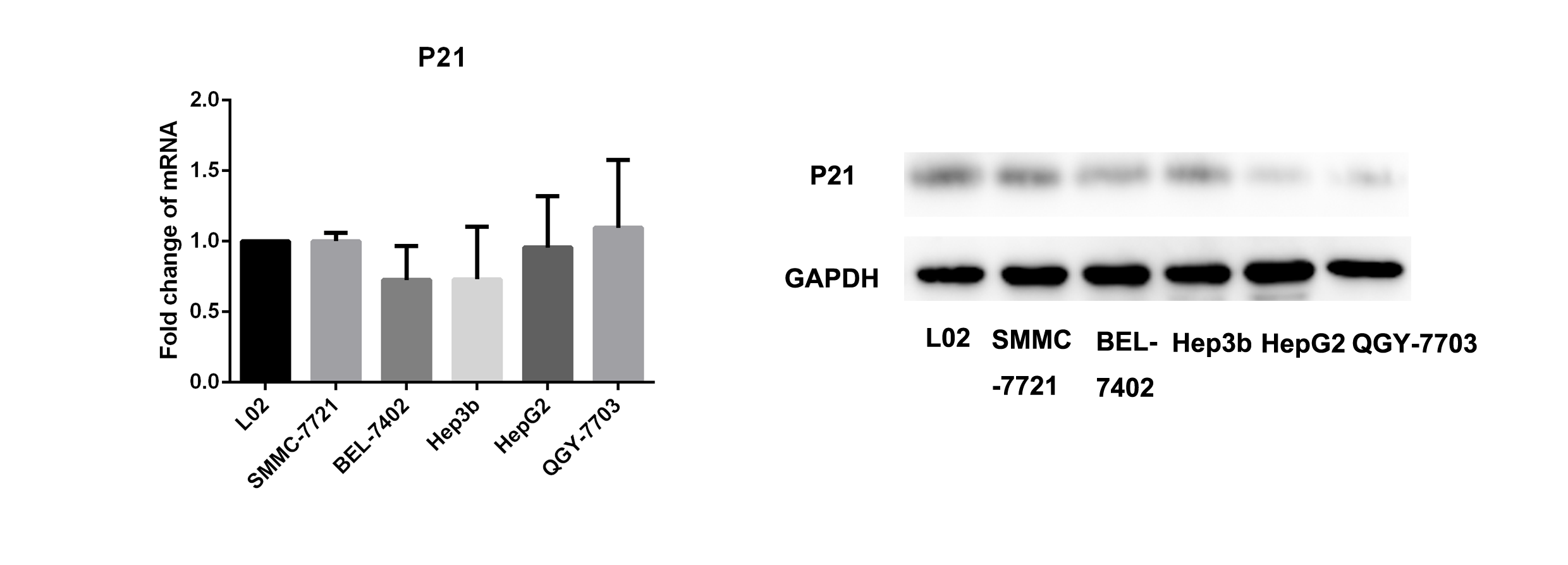

Supplement: Supplementary file 2 — Additional file 2: Figure S2. The expression of let-7b in hepatocyte and HCC cell lines. RT-PCR and western blot were performed to detect let-7b expression in normal liver cells and HCC cell lines. Data from three replicates are shown as the means (± SD). [file 13578_2020_443_MOESM2_ESM.tif]
